# Docking Simulation of the Binding Interactions of Saxitoxin Analogs Produced by the Marine Dinoflagellate *Gymnodinium catenatum* to the Voltage-Gated Sodium Channel Na_v_1.4

**DOI:** 10.3390/toxins8050129

**Published:** 2016-05-06

**Authors:** Lorena M. Durán-Riveroll, Allan D. Cembella, Christine J. Band-Schmidt, José J. Bustillos-Guzmán, José Correa-Basurto

**Affiliations:** 1 Departamento de Plancton y Ecología Marina, Centro Interdisciplinario de Ciencias Marinas–Instituto Politécnico Nacional, La Paz, B. C. S. 23096, Mexico; cbands@ipn.mx; 2 Alfred-Wegener-Institut, Helmholtz Zentrum für Polar-und Meeresforschung, Bremerhaven 27570, Germany; allan.cembella@awi.de; 3 Centro de Investigaciones Biológicas del Noroeste, La Paz, B.C.S. 23201, Mexico; jose04@cibnor.mx; 4 Laboratorio de Modelado Molecular y Diseño de Fármacos, Escuela Superior de Medicina–Instituto Politécnico Nacional, Mexico City 11340, Mexico; jcorreab@ipn.mx

**Keywords:** voltage-gated sodium channel, benzoyl saxitoxin analogs, molecular docking, binding affinity

## Abstract

Saxitoxin (STX) and its analogs are paralytic alkaloid neurotoxins that block the voltage-gated sodium channel pore (Na_v_), impeding passage of Na^+^ ions into the intracellular space, and thereby preventing the action potential in the peripheral nervous system and skeletal muscle. The marine dinoflagellate *Gymnodinium catenatum* produces an array of such toxins, including the recently discovered benzoyl analogs, for which the mammalian toxicities are essentially unknown. We subjected STX and its analogs to a theoretical docking simulation based upon two alternative tri-dimensional models of the Na_v_1.4 to find a relationship between the binding properties and the known mammalian toxicity of selected STX analogs. We inferred hypothetical toxicities for the benzoyl analogs from the modeled values. We demonstrate that these toxins exhibit different binding modes with similar free binding energies and that these alternative binding modes are equally probable. We propose that the principal binding that governs ligand recognition is mediated by electrostatic interactions. Our simulation constitutes the first *in silico* modeling study on benzoyl-type paralytic toxins and provides an approach towards a better understanding of the mode of action of STX and its analogs.

## 1. Introduction

Saxitoxin (STX) and its numerous naturally occurring analogs are alkaloids with neurotoxic properties. These toxins are hetero-tricycles with two guanidinium groups that are positively charged at physiological pH (~7). The guanidinium groups are responsible for toxin polarity and water solubility, and are essential for sodium channel binding properties. These toxins also have two hydroxyl groups at the C-12 position that are critical for binding to the sodium channel [1].

Intoxication by these toxins can be severe and occasionally fatal for consumers of contaminated shellfish [2]. Typically, the toxins affect humans after consuming bivalve shellfish that have ingested toxic dinoflagellates during a harmful algal bloom, thus they are known colloquially as paralytic shellfish toxins (PSTs), but rare cases of poisoning can also occur from eating contaminated fish or crustaceans [3].

In the marine environment, PSTs are produced by free-living dinoflagellates belonging to three genera [4]. These toxigenic dinoflagellates include about a dozen species of the gonyaulacoid genus *Alexandrium*, plus the heavily armored species *Pyrodinium bahamense* (*P. bahamense*) and the naked chain-forming *Gymnodinium catenatum* (*G. catenatum*), the only PST-producing gymnodinoid dinoflagellate [5].

At least 57 naturally occurring PST analogs have been identified in diverse aquatic and terrestrial organisms [6,7]. These analogs have been subdivided according to their chemical substituent groups on the lateral chain, as carbamoyl, decarbamoyl, *N*-sulfocarbamoyl, or benzoyl (*p*-hydroxybenzoyl, di-hydroxybenzoyl, or sulfobenzoyl) derivatives. Variation in the substituent groups confers different toxin potency in mammalian systems, ranging in relative toxicity from the most potent, STX, to the least potent, *N*-sulfocarbamoyl derivatives, usually determined by intraperitoneal injection into laboratory mice [8].

Benzoyl toxins (also called GC toxins) have been exclusively identified in strains of *G. catenatum* [9,10,11,12,13]. Chemical characterization and confirmed identification of these analogs has been limited, mainly due to the lack of analytical standards [14]. Currently, three structures have been unequivocally determined by nuclear magnetic resonance (NMR) [9] and another 15 putative structures have been proposed based upon mass spectrometry [11,15], but the specific toxicity of these putative analogs is still unknown.

Voltage-gated sodium channels (Na_v_), the main target for PSTs, are present in excitable cell membranes. The voltage-gated channel proteins provide the main current underlying rapid signal propagation, and consist of a ~230 kD α-subunit that forms the pore with the selectivity filter, where the binding sites for multiple drugs and toxins are found [16]. The domains are thought to be organized circumferentially around the ion-conducting pore. The amino acid chains that line the ion permeation pathway are known as the P-loops [17]. A water-filled region encompassed by the four P-loops forms the outer vestibule of the channel. The outer vestibule is the region of the α-subunit that includes the receptors for site 1 sodium channel blockers such as tetrodotoxin (TTX) and STX [16].

Given the lack of information on toxicity and mode of action on their biological target of these recently discovered GC analogs, theoretical studies are essential to decipher the recognition and binding patterns. Studies with selected STX analogs have shown strong binding affinities to the Na_v_ channel [18]. As there is no mammalian model for this voltage gated channel, homology models have been proposed. These models are based on the crystal structures of bacterial Na_v_ channels, and help to interpret and understand their functional mechanisms. Such models are useful in structure-based design of therapeutics [19] and, in the present case, to gain knowledge about specific toxicity of derivatives, such as benzoyl STX analogs that have not been subjected to *in vivo* trials.

The PSTs are selective blocking agents that reduce functional Na_v_ channels, while occupying a site near the channel opening with a 1:1 affinity in the binding site 1 [20,21]*.* STX and its analogs are proposed [22] to bind to the same site on Na_v_ because of their similar chemical structures, but with different affinities. Cloning the family of mammalian Na^+^ channel genes has led to identification of four key residues in the selectivity region known as an aspartate-glutamate-lysine-alanine (DEKA) motif. This motif consists of one amino acid from each of the four P-loop regions from domains I–IV, respectively, Asp400 (D400), Glu755 (E755), Lys1237 (K1237), and Ala1529 (A1529) [21].

Current docking programs consider the protein as a rigid body and the ligand as a flexible molecule, which reduces the computational cost by omitting conformational changes that occur in protein molecules due to ligand binding [23]. Although such proteins may be in fact somewhat flexible, such docking simulations are very useful for prediction of ligand-protein interactions, providing an opportunity to explore recognition properties and identify potential pharmacophores [24]. As this is the first attempt to apply molecular tools in assessing channel binding properties and consequent toxicity of recently discovered toxin analogs, namely benzoyl derivatives, these docking simulations are a suitable tool for a first approach.

With scarce information on the recognition of STX and its analogs at the channel binding site, we performed molecular docking studies with two alternative Na_v_1.4 models, to depict the kinds of interactions that govern their recognition. Our hypothesis is that the guanidinium groups with positive charges and sulfate groups with negative charges engage in electrostatic interactions with basic or acidic residues located in the Na_v_ recognition site.

## 2. Results

### 2.1. Interaction of STX and Analogs with Na_v_1.4 Amino Acid Residues

All analogs interacted with the Na_v_1.4 residues with both models, but to a different extent. The results of these interactions are explained for each model in the following sections.

#### 2.1.1. Na_v_1.4 Model 1

**Figure 1:**
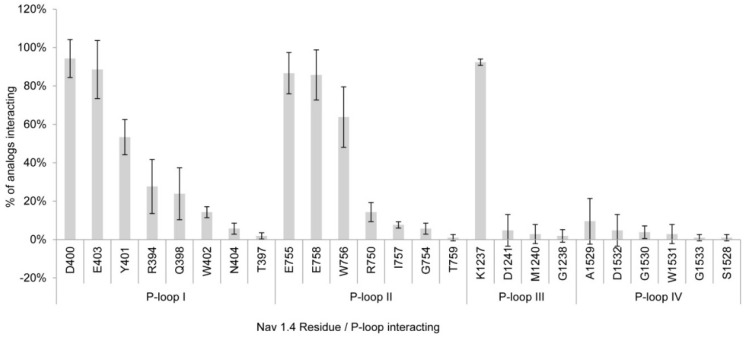
Residues and P-loops involved in binding interaction with saxitoxin(STX) and its analogs in Na_v_1.4 Model 1. Percentages represent the average number of residues in the three dockings studies.

**Figure 2:**
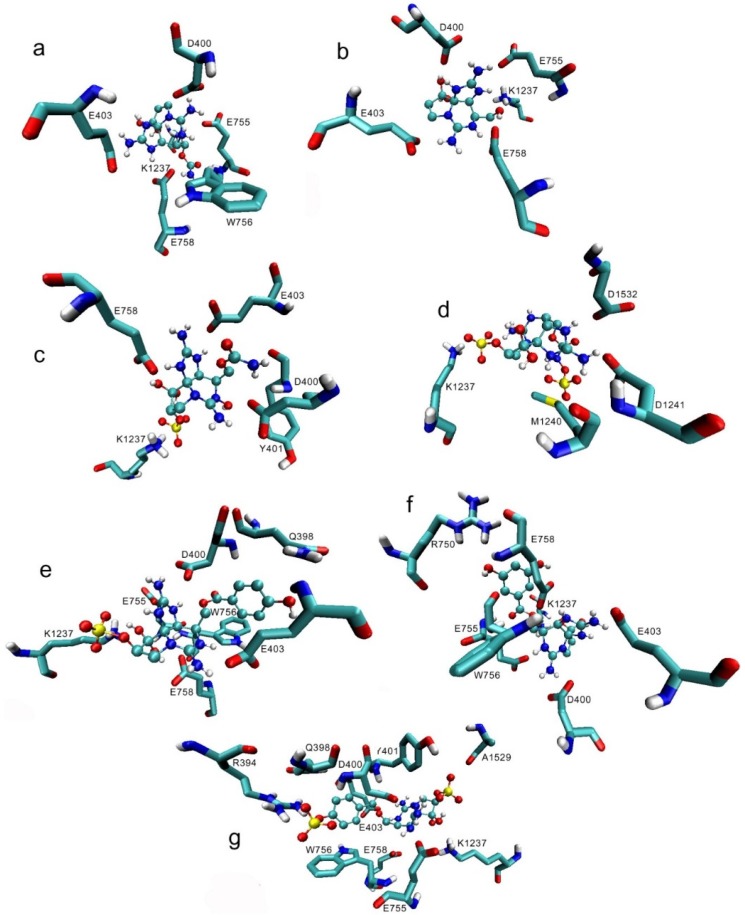
Binding of saxitoxin (STX) and some of its analogs with the residues from the Na_v_1.4 Model 1 according to this docking study: (**a**) STX; (**b**) decarbamoyl saxitoxin (dcSTX); (**c**) gonyautoxin (GTX4); (**d**) C2 toxin (C2); (**e**) *p*-hydroxybenzoyl (GC1); (**f**) di-hydroxybenzoyl (GC3a); and (**g**) sulfobenzoyl (GC1b) toxins. Spheres = toxins; sticks = Na_v_ residues. These toxins represent those that have the lowest ΔG values per family of toxins and for which the main non-bond interactions between guanidinium and E403, D400, E258, as well as between sulfate groups and K1237 and R394, are electrostatic.

The three independent docking simulations showed an average of 22 and a maximum of 25 Na_v_ 1.4 channel residues interacting with the toxin analogs. The residues that showed interactions with >80% of the PST analogs were D400, K1237, E403, E755, and E758 (Figure 1). Of these residues, D400, E755, and K1237 correspond to the DEKA selectivity filter ring, along with A1529, which showed much less interaction, whereas E403 and E758 are part of the outer ring possessing a negative charge. Most of the residues involved in binding to the toxins belong to P-loops I and II (eight and seven residues, respectively); these residues interacted with more toxins than the residues from P-loops III and IV, except for K1237 in P-loop III, which interacted with 92.4% of the toxins in the docking simulations. Figure 2a–g shows that STX and some of its analogs reach the side-chain with their guanidinium group of some residues (E403, D400, E755) by electrostatic interactions, whereas there are hydrogen bonds with both hydroxyl groups located at C-12.

#### 2.1.2. Na_v_1.4 Model 2

**Figure 3:**
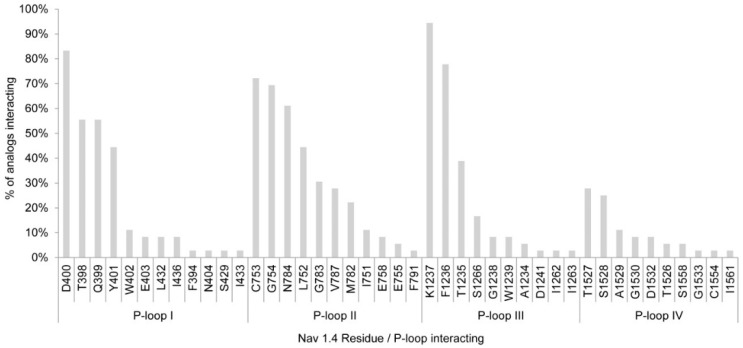
Residues and P-loops involved in binding interaction with saxitoxin and its analogs in Na_v_1.4 Model 2.

According to Model 2, PST analogs showed more interactions with the Na_v_1.4 channel residues than for the initial model. This docking simulation showed interactions with 43 residues rather than the maximum 25 residues with Model 1. Only two residues interacted with >80% of the toxin analogs, namely D400 and K1237, both from the DEKA selectivity filter ring, whereas E755 and A1529, also part of the DEKA ring, showed interaction with <20% of the analogs in Model 2 (Figure 3).

**Figure 4:**
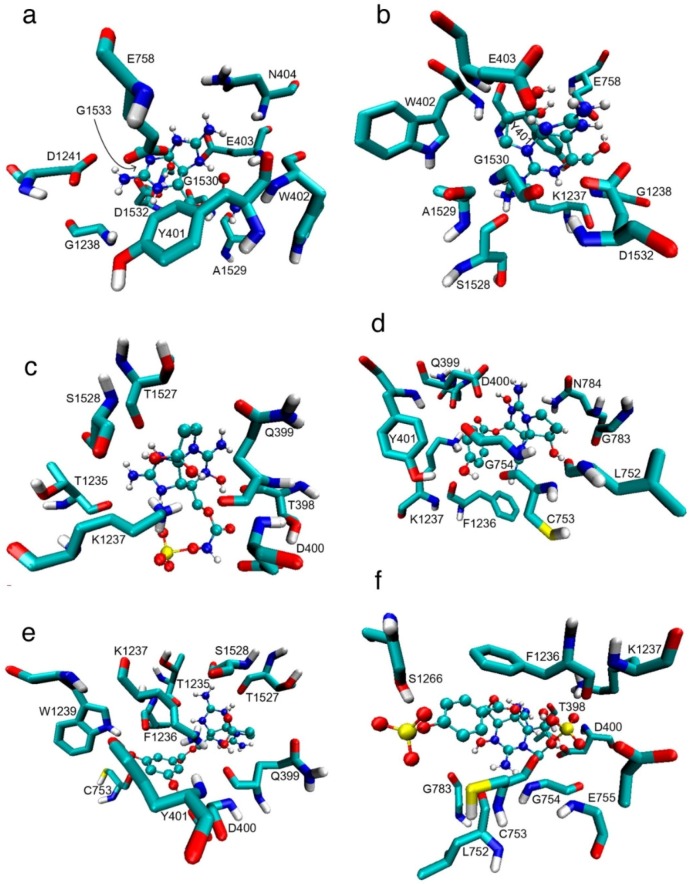
Binding of STX and some of its analogs with the residues from the Na_v_1.4 Model 2 according to this docking study: (**a**) STX; (**b**) decarbamoyl saxitoxin (dcSTX); (**c**) B toxin (B2); (**d**) *p*-hydroxybenzoyl (GC6); (**e**) di-hydroxybenzoyl (GC3a); and (**f**) sulfobenzoyl (GC5b). Spheres = toxins; sticks = Na_v_ residues. These toxins represent those that have the lowest ΔG values per family of toxins and for which the main non-bond interactions are electrostatic, e.g., between guanidinium and E403 and D400, except for GC3a, as well as between sulfate groups and K1237, except for GC5b.

The residues that showed interactions with between 60% to 80% of the toxins were C753, G754, N758 and F1236; all of these residues belong to P-loops II and III. Figure 4a–f also shows that, according to this model, STX and some analogs reach the side chain with their guanidinium group by electrostatic interaction and that the hydroxyl group at C-12 forms hydrogen bonds with the residues.

#### 2.1.3. Comparison between Model 1 and Model 2

**Figure 5:**
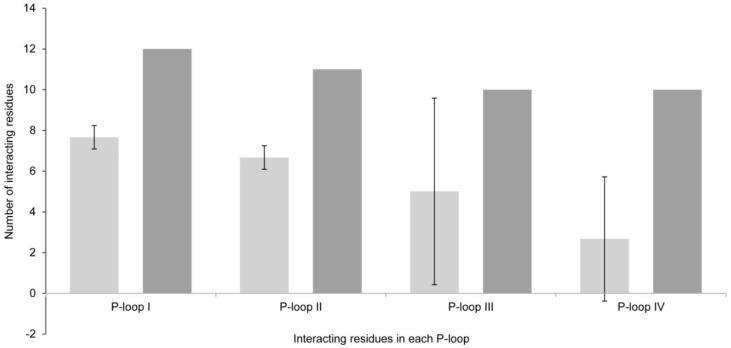
Total number of interacting residues in Model 1 (light gray) *versus* Model 2 (dark gray) in each P-loop.

**Figure 6:**
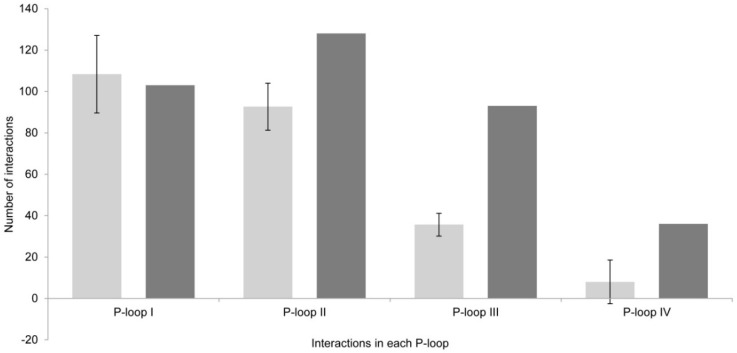
Total number of interactions per P-loop in the two models. Model 1 (light gray) and Model 2 (dark gray).

There are great differences between the results obtained via Model 1 *versus* Model 2, even when the differences in the residues that form these two channel models are not apparently very large. In Model 1, there was an average of 22 channel residues interacting with all toxin analogs (Figure 5), whereas in Model 2, 43 residues (almost double in number) interacted with all tested PST analogs. These differences were also visible in the P-loops of each domain (Figure 6).

The total interactions, *i.e.*, the sum of all interactions of all analogs with all residues, were greater with Model 2 as well. In Model 1, there was an average of 247 interactions, whereas Model 2 indicated a total of 360 interactions (47% more).

### 2.2. Binding Free Energy

#### 2.2.1. Na_v_1.4 Model 1

**Figure 7:**
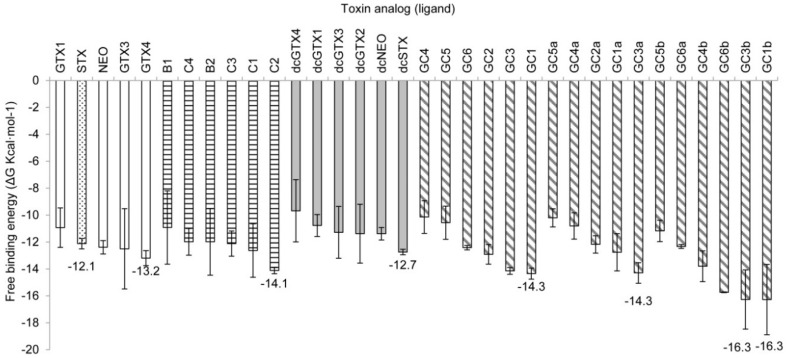
Free-binding energy (ΔG, kcal·mol^−1^) of the docked toxins with Model 1. Toxins are grouped according to their lateral chain: empty bars, carbamoyl toxins; horizontal hatching, *N*-sulfocarbamoyl toxins; gray bars, decarbamoyl toxins; diagonal hatching, benzoyl toxins. The most potent PST, STX, is shown in the dotted bar. Numbers indicate the lowest ΔG in each group compared with that of STX.

This model exhibited high affinity values for STX and some of its analogs, ranging from −7.3 to −16.3 kcal·mol^−1^. The sulfobenzoyl derivatives GC3b and GC1b showed unexpected high ΔG values of −16.3 kcal·mol^−1^. The known most potent analogs, STX, neosaxitoxin (NEO), and decarbamoyl saxitoxin (dcSTX), which have relative toxicities of ~1 [25] showed similar average ΔG values between −12.1 and −12.7 kcal·mol^−1^ (Figure 7). For each structural family, the ligands that had the lowest ΔGs were GC3b/GC1b < GC3a/GC1 < C2 < GTX4 < dcSTX.

#### 2.2.2. Na_v_1.4 Model 2

**Figure 8:**
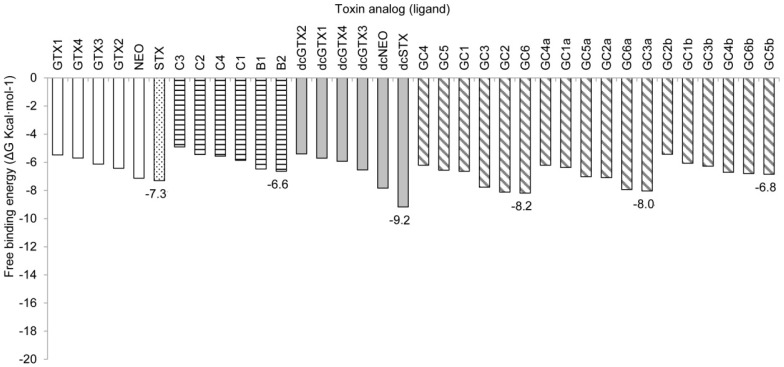
Free-binding energy (ΔG, kcal·mol^−1^) of the docked toxins with Model 2. Toxins are grouped according to their lateral chain: empty bars, carbamoyl toxins; horizontal hatching, *N*-sulfocarbamoyl toxins; gray bars, decarbamoyl toxins; diagonal hatching, benzoyl toxins. The most potent PST, saxitoxin (STX), is shown in the dotted bar. Numbers indicate the lowest ΔG in each group compared with that of STX.

In this model, the free binding energy values were higher in general, ranging from −9.17 to −4.91 kcal·mol^−1^, with the lowest ΔG value corresponding to the analog dcSTX, followed by GC6, GC2, GC3a, GC6a, decarbamoyl neosaxitoxin (dcNEO), GC3, STX, and NEO, analogs that had the lowest binding energy values, with ΔGs ranging from −8.19 to −7.13 kcal·mol^−1^. In this case, the known most potent analogs (STX, NEO and dcSTX) showed (as expected) a lower ΔG value in comparison with the less potent analogs, but along with these well-known toxic molecules, some GC toxins of unknown toxicity showed low energy values as well (Figure 8). For each structural family, the analogs showing the lowest ΔG values were dcSTX < GC6 < GC3a < STX < GC5b < B2.

## 3. Discussion

Despite knowledge of the basic structures and origin of the benzoyl STX analogs for over two decades [9], there have been few targeted efforts to determine associated properties, including mammalian toxicity, receptor-binding affinities and biological action mechanisms. These challenges remain unmet because of the lack of a comprehensive suite of analytical standards, which in turn is a function of the difficulty in their isolation and purification from *G. catenatum* cultures.

The alternative strategy to explore these properties was to use computational tools, including structural analysis to decipher their binding recognition properties and to explore their non-bonding interactions and ∆G values. Molecular docking studies of PSTs have already proven successful for exploring and interpreting hindrance, electrostatic, and complementary shape properties between receptors and ligands, and have also provided ∆G values and binding poses [26]. The docking modeling approach has the potential to reveal, in great structural detail, the molecular interactions, mechanisms, and structural contacts involved in binding recognition, which are not easily deduced from electrophysiological experiments [27]. Previous computational studies have been able to reproduce many of the experimental observations, including the binding affinity and specificity of a given ligand, such as a toxin analog, in several subfamilies of ionic channels [27]. Docking simulations allow fast screening of many ligands for a given protein [28,29], although their accuracy is limited [30]. From these studies, it is possible to obtain important information about the mode of action and the affinity for the ligands, which helps explain, at least in part, the toxicity of diverse molecules, prior to *in vivo* or *in vitro* testing*.*

Some computational studies of Na_v_ channel-binding affinities of pore-blocking toxins have accessed homology models to build the channel, as well as molecular docking simulations for theoretical validation. Models of the mechanism and binding modes of pore-blocking toxins have been proposed, but the exact binding modes between toxins and Na_v_ channels have not yet been elucidated [27].

Free binding energy (ΔG, kcal·mol^−1^) values given by these simulations are the result of ligand-residue interactions, and thus serve as an intermolecular affinity indicator. The ΔG is the sum of non-covalent interactions, as well as the planar and dihedral angles, and atomic bonds, all physical properties described elsewhere in the force fields, and which make the most energetically important contribution. The lowest ΔG values indicate the highest affinity between the ligand and the protein [31]. Since the ΔG of the ligand-protein complex is the ultimate determinant of binding affinity, predicting this value is the most important goal of the theoretical and computational studies.

### 3.1. Interactions with Na_v_ Residues

Previous studies showed the importance of the acidic residues to the binding properties, whereby carboxylic acid side chains are located in the same structural positions (toward the channel) in the four domains to which STX binds [21,32]. In our study, in the simulation with Model 1, we observed the interaction of STX and its analogs with the side chain D400 and K1237 at >90%. With other acidic residues, E403, E755, and E758, the frequency of interaction was >80% (Figure 1). The residue K1237 was the second most accessed, with 92% of all the analogs interacting with this residue. Only residue D400 had a slightly higher percentage (94%) of interactions with STX and analogs (Figure 1). Where the sulfate group is absent, the main non-bond interactions are between the guanidinium group and acidic residues, creating electrostatic interactions, whereas for those STX analogs where the sulfate group is present (GTX4, C2, GC1, and GC1b), the main non-bond interactions are between the sulfate group and basic residues, again yielding electrostatic interactions.

With Model 2, D400 and K1237 were also the residues with the most interactions, with >80% of the analogs interacting with them. The other residues of the DEKA ring, however, showed less interactions than in Model 1, with F1236 and C753 the most accessed residues after D400 and K1237 (78% and 76% of the analogs interacting, respectively) (Figure 3). Even though there were fewer interactions with the DEKA ring residues, the analogs interacted with a greater diversity of residues. As depicted for Model 1, the main non-bond interactions are between the guanidinium group and acidic residues, whereas for STX analogs where the sulfate group is present (*i.e.*, B2) the main non-bond interactions are between the sulfate group with basic residues. In both cases, these interactions are electrostatic, whereas for GC5b hydrogen bonds are generated with S1266 (Figure 4).

Based on these binding pose data, we postulate that STX and its analogs access the Na_v_1.4 primarily via electrostatic interactions, which are dominant (lowest ∆G values) among the non-covalent interactions between ligands and proteins [33]. These kinds of interactions could also explain the high affinity and relative toxicities of these PSTs, as has been demonstrated for other toxins [34].

### 3.2. Free-Binding Energy and Specific Toxicity

In Model 1, ΔG of the most potent analog according to mammalian intraperitoneal bioassays (Table 1), STX, was −12.1 kcal·mol^−1^, similar to or even greater than that of other STX analogs. In comparing free energy values of toxins with markedly different toxin potency, such as the *N*-sulfocarbamoyl C2 (ΔG −14.1 kcal·mol^−1^) *versus* STX, it is important to note that its molar toxicity is about 1/10 of that of STX (Table 1). This difference could be caused by the higher molecular weight of C2 compared with STX, and hence a larger surface interaction in the simulation with Na_v_1.4. Under physiological conditions, the higher molecular weight of C2 could also affect its passage through lipid-protein barriers thereby diminishing its toxic properties. Alternatively, its lower toxicity could be the result of the compensated charges due to the two sulfate groups with negative charges, making this molecule electrochemically neutral, whereas for STX the lack of sulfate groups leaves its positive guanidinium charges uncompensated.

From the toxin analogs that showed lower ∆G values with Model 1 (GTX4, C2, dcSTX, GC1, GC3a and GC1b) (Figure 7), four have the double-negatively charged sulfate moiety at R2 or R3 (See Materials and Methods). The negative charge could therefore increase the affinity by electrostatic interactions with K1237 in the simulation.

In the simulation with Model 2, the overall ∆G values were higher, but these values showed slightly better accordance with the actual potency of the analogs that have been subjected to *in vivo* toxicity tests, as shown in Table 1. In this simulation, the analogs with lower ∆G were those with higher toxicity, such as STX and dcSTX (∆G values = −7.31 and −9.17 kcal·mol^−1^, respectively), and toxin B2 (∆G value = −6.64 kcal·mol^−1^). Though the differences among ∆G values of highly potent toxins STX and dcSTX and the less potent B2 is small in our simulation, we found that this model shows a slightly better correspondence than with Model 1. According to this Model 2 simulation, the GC analogs with lowest ∆G values, GC6, GC3a and GC5b, could be then the most toxic analogs of this toxin family. In their docking simulations, Choudhary and coworkers [32] found that the gonyautoxins (GTX), the C-11 sulfated STX analogs, were approximately three-fold less potent than their non-sulfated counterparts. Furthermore, the presence of N-1-OH conferred better binding to the Na_v_ channel with a sulfate group at C-11 (e.g., GTX1,4) than when this group is absent from this site (e.g., NEO). Significantly, they found that a sulfate group at C-11 interacted mainly with the acidic residues E758 and D1241.

In our study, in the simulation with Model 1, we found a lower binding energy for non-sulfated C-11 toxins than for sulfated C-11 toxins, a difference of −1.2 kcal·mol^−1^. With Model 2, there were no differences between C-11 sulfated and non-sulfated analogs. We did find that the sulfate group creates electrostatic interactions with K1237. It is important to consider the molecular dimensions in the context of blocking efficacy and binding affinities that are dependent upon charge- and steric hindrance-effects. For example, the structural dimensions of the STX crystal are 8.5 × 4 × 6.5 Å, which is increased by almost 6 Å along the major axis in benzoyl analogs. Despite the larger size of the benzoyl analogs, they do fit into the Na_v_ central cavity, as observed in *in vitro* studies [1,18]. Hence, this cavity is large enough to host these toxins.

Chen and Chung [37] found that the peptide μ-conotoxin PIIIA in the outer vestibule of the voltage-gated sodium channel Na_v_Ab is capable of blocking the channel by various binding modes, despite its large molecular size (over 20 amino acids, ~2500 Da). The same mechanisms are plausible for the STX analogs, which are smaller molecules (~300–569 Da) than the peptide toxins, thereby allowing more free movement in the Na_v_ channel, and reaching positively or negatively charged amino-acid residues. With this docking study we were able to demonstrate different binding modes for toxins with similar free binding energies, and not a single mode, as is generally assumed, and that alternative binding modes were equally probable.

All STX analogs bind to the same site, although with different binding modes and affinities [22]. These theoretical observations were confirmed in our study. However, we found no direct relationship between binding affinity and toxin potency, likely because biological properties that influence pharmacokinetic parameters, including the quantity of molecules reaching the targets after crossing biological barriers, are not considered in docking studies. The molecular movements in aqueous systems or within lipid membranes are dependent upon atomic charge interactions capable of forming hydrogen bonds with water molecules [38]. The target molecules vary according to their substituents, which affect ligand recognition and also influence their biological availability, and consequently, the quantity that reaches the protein target. These factors have to be considered when relating chemical properties with the biological (toxicity) aspects of the STX analogs.

Llewellyn [39] performed a study of quantitative structure-relationship activity (QSAR) to use molecular descriptors to describe the potency of known natural toxins and predict the toxicity of untested natural STX analogs. This study [34] did not consider the binding properties, which could yield differential affinity values related to blocking potency and, consequently, toxicity effects. Nevertheless, he found a relationship between the molecular descriptors and toxicity properties of PSTs and evidence linking sodium channel affinity of PSTs and toxicity, even though the relationship was not linear or direct and differed among the different sodium channel isoforms. This variation occurs because each sodium channel isoform has different residues that form the channel tunnel, thereby affecting toxin recognition.

Our docking study provides critical information in clarifying the binding mechanism of STX and its analogs on the Na_v_1.4 on two different conformations, determining the binding poses and ∆G values, without experimental assays. In rat synaptosomes, benzoyl STX analogs strongly bind to the channels, with less potency than STX [18]. In our docking simulations, we found that 12 benzoyl analogs bound strongly to the Na_v_1.4 channel according to Model 1, and five analogs with Model 2, all of them having a lower ΔG than STX (Figure 7 and Figure 8). Again, the absence of direct correlation between ΔG and toxin potency could result from not considering external biological parameters that modify channel behavior, such as water and lipid components. Such factors are associated with pharmacokinetic behavior and ligand recognition processes, including molecular movements that are not considered in docking studies. Protein movement is important, as depicted in this docking study, for which Model 1 and Model 2 showed somewhat different but not incompatible docking results, with prominent electrostatic interactions, despite interactions with different residues in either model.

It is important to bear in mind that there is a huge difference between voltage-gated sodium channel binding and whole animal toxicity. Furthermore, our simulation was performed with only one of the many types of Na_v_ channels, and, as has been recently demonstrated for several Na_v_ human channels, the response to STX and its analogs is channel-type dependent [40]. We recognize that although there are major biological factors that are not taken into account by docking simulations, this approach can describe comparative binding properties of toxins on the Na_v_ channel at the atomic level with approximate free energy values. These data can be compared with toxicity data as analytical standards for novel toxin analogs become available for structural and potency analysis.

## 4. Conclusions

Even with the lack of experimental data on toxicity for several novel and poorly known STX analogs, including benzoyl variants produced by *G. catenatum*, our simulations of binding behavior predict that at least some of them would be toxic because they reach key residues by electrostatic interactions. This poses challenges for human health protection, where blooms of *G. catenatum* occur in waters from which shellfish are harvested, because these compounds are not subject to routine analysis and quantitative toxin risk remains unknown. Although our *in silico* studies do not show a linear relationship between theoretical and experimental data on toxin affinities for the Na_v_ channel and biological potencies, our simulation constitutes the first modeling of benzoyl-type paralytic toxins. Our simulations describe the main interactions that could define the relationship of the molecule to its specific toxicity, although better Na_v_ models or different *in silico* techniques, with reference to molecular dynamics or quantitative structure–activity relationships, are required to define linear correlations to assess and predict specific toxicity of these analogs in a more reliable way. In any case, this docking simulation approach contributes to a better understanding of the mode of action of STX and its analogs, and indicates that the principal binding mechanism that governs ligand recognition is mediated by electrostatic interactions.

## 5. Materials and Methods

### 5.1. Voltage-Gated Sodium Channel Models

**Figure 9:**
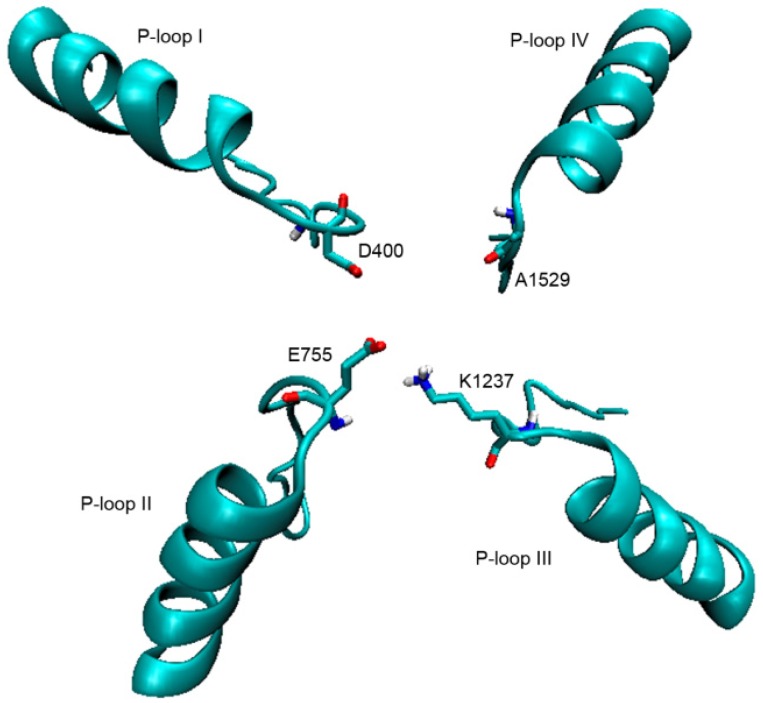
Model 1; P-loops from domains I–IV. The DEKA motif residues are drawn in sticks.

The first model (dubbed Model 1) of the Na_v_1.4 outer vestibule (Robert J. French, Department of Physiology & Pharmacology, University of Calgary, Calgary, Canada, personal communication) was generated following the approach of Lipkind and Fozzard [41]. This channel building method employs the S5 and S6 backbone coordinates and the pore helices from the KcsA bacterial potassium channel and is supported with functional biophysical data [16]. This model was validated by exploring the recognition of tetrodotoxin (TTX), 11-SO_3_-STX, and μ-conotoxins at binding site 1, which is located in the α subunit of the channel [16]. Here, we used this model to perform the docking simulations in triplicate to obtain average values and standard deviations of all energy calculations (Figure 9).

The second model (Na_v_1.4 Model 2), proposed by Mahdavi and Kuyucak [19], was also constructed by homologation with the crystal structure of the bacterial Na_v_, and validated with functional data for binding of μ-conotoxin GIIIA. Both models have been applied for simulations with μ-conotoxin GIIIA, and subjected to molecular dynamic analysis, yielding good results with the conotoxin.

**Figure 10:**
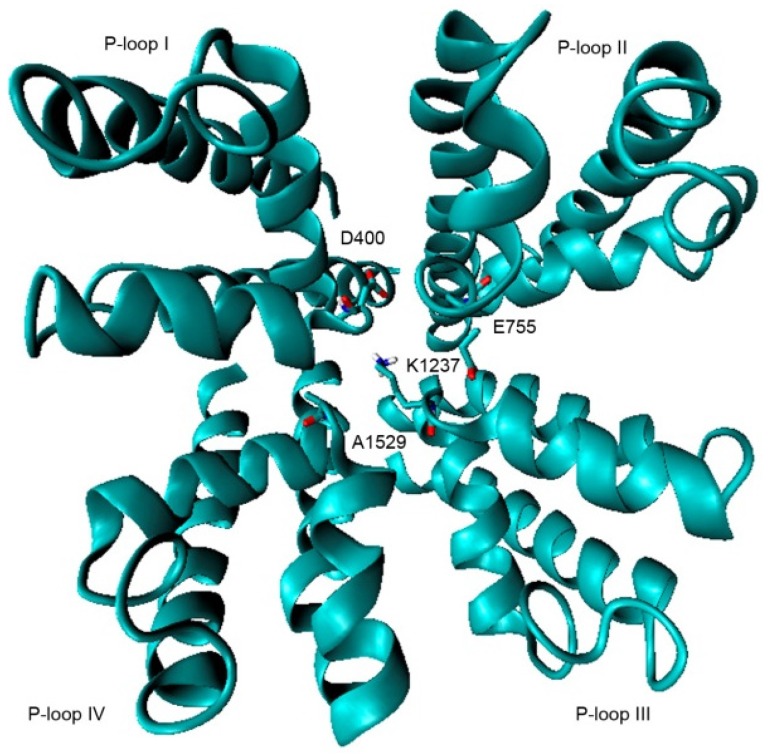
Model 2; P-loops from domains I–IV. The DEKA motif residues are drawn in sticks.

Model 2 is slightly more complex than Model 1, although there are no major differences between them. We used this latter refined model for one docking simulation to compare results with those generated from Model 1 but not for strict statistical comparison (Figure 10).

The alignments used for both models and their differences are shown in Table 2.

### 5.2. Molecular Docking Simulations

**Figure 11:**
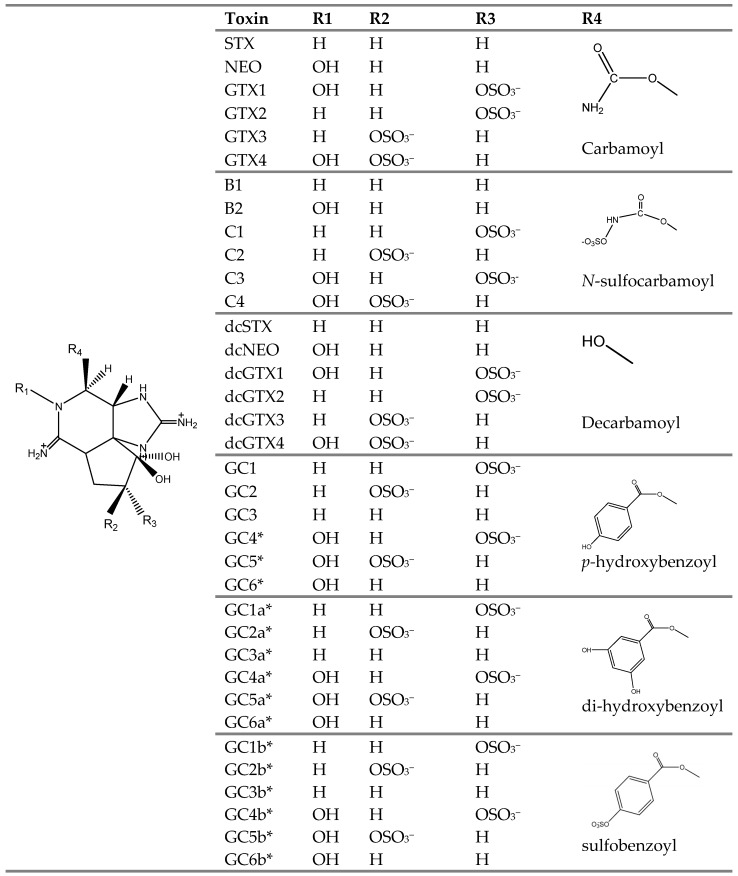
Molecular structures of STX and its analogs produced by *G. catenatum*. All of the analogs depicted were subjected to *in silico* modeling simulations in our study. Structures marked with * have only been inferred from mass spectrometry analysis [11] but are assumed to be correct for docking simulations.

Docking simulations were based upon the proposed structures of STX and analogs (Figure 11), some of which have not been structurally confirmed, but rather inferred from mass spectrometry analysis [11]. We drew the structures of STX and analogs according to the reported stereochemistry [15].

The tridimensional structures of ligands were developed with GAUSSVIEW 5.0 software [42]. We obtained the conformation associated with the lowest energy and the highest stability by structural optimization at the AM1 semi-empirical level by GAUSSIAN 3.0 software [43]. Once the structures were energetically stable, we performed the docking simulations with AUTODOCK 4.0.1. [44]. In the docking simulations, the protein was kept rigid and the docked ligands were considered flexible. The grid box was set at 60 × 60 × 60 Å^3^ and centered on the channel orifice, considering the external vestibule of the Na_v_1.4 α-subunit, where binding site 1 is located [45]. We chose the Lamarckian genetic search algorithm for the best ligand conformation, and considered a set of 100 best conformers for each ligand. With Na_v_1.4 Model 1, we ran the docking simulations in triplicate, each time with a different computer, thereby obtaining 300 simulations for each ligand (10,500 simulations). We calculated an average of the lowest three free binding energy (∆G) values per ligand. For comparison with Na_v_1.4 Model 2, we ran only one docking simulation, but again considered a set of 100 best conformers for each ligand. Molecular docking results were analyzed with AUTODOCK TOOLS 1.5.6. [46] and figures were created with VMD 1.9.1 software [47].

## Figures and Tables

**Table 1:** Relative acute toxicities of saxitoxin and derivatives according to intraperitoneal mouse bioassays, modified from Munday (2014) [25]. Relative values calculated from * Sullivan *et al.*, (1985) [35] and ** from Oshima (1995) [36].

Toxin Analog	Relative Toxicity
STX	1.0
NEO	1.0
GTX1	1.0
GTX2	0.4
GTX3	0.6
GTX4	0.7
dcSTX	1.0
dcGTX1	0.5 *
dcGTX2	0.2
dcGTX3	0.4
dcGTX4	0.5 *
dcNEO	0.4
B1	0.1
B2	0.1
C1	0.01 **
C2	0.1
C3	0.01 **
C4	0.1

**Table 2:** Alignments of P-loops from domains I–IV used for each model. Differences between the two models are noted in red. The DEKA residues in the four domains forming the selectivity filter are highlighted. Modified from [16,19].

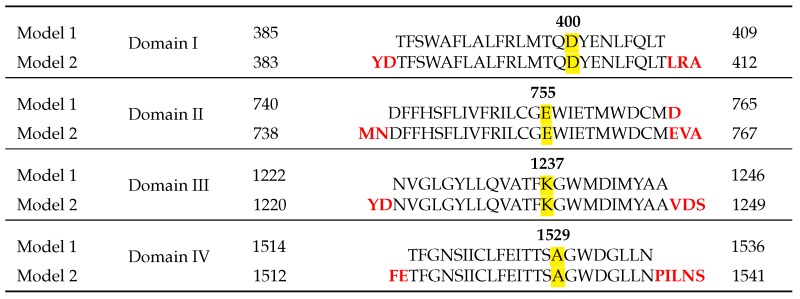

## Abbreviations

The following abbreviations are used in this manuscript:

Na_v_: voltage-gated sodium channel
PSTs: paralytic shellfish toxins
PSP: paralytic shellfish poisoning
TTX: tetrodotoxin
